# Flow Cytometry: A Novel Approach for Indirect Assessment of Protamine Deficiency by CMA3 Staining, Taking into Account the Presence of M540 or Apoptotic Bodies

**Published:** 2011-12-22

**Authors:** Zohreh Fathi, Marziyeh Tavalaee, Abbas Kiani, Mohammad Reza Deemeh, Mehrdad Modaresi, Mohammad Hossein Nasr-Esfahani

**Affiliations:** 1 Department of Reproduction and Development, Reproductive Biomedicine Center, Royan Institute for Animal Biotechnology, ACECR, Isfahan, Iran; 2 Payame Noor University, Isfahan, Iran; 3 Isfahan Fertility and Infertility Center, Isfahan, Iran; 4 Islamic Azad University-Khorasgan Branch, Isfahan, Iran; 5 Department of Embryology, Reproductive Biomedicine Research Center, Royan Institute for Reproductive Biomedicine, ACECR, Tehran, Iran

**Keywords:** Flow Cytometry, Sperm, Protamine, Merocyanine

## Abstract

**Background:**

Chromomycin A3 (CMA3) staining, either by the slide method or fluorescence microscopy, is widely used for indirect assessment of protamine deficiency in a semen sample. Flow cytometry is the most suitable tool to improve assessment accuracy, both in terms of statistical analysis and for prevention of observer variation. This study provides a simple procedure to account for merocyanine 540 (M540) or apoptotic bodies, which result in underestimation of the percentage of CMA3 positivity, by using propidium iodide (PI) staining. Therefore, this study aims to evaluate the percentage of CMA3 by PI staining to exclude M540 bodies that prevent underestimation of CMA3 staining.

**Materials and Methods:**

This study is an experimental study. Semen samples collected from 104 infertile men who referred to the Andrology Unit of the Isfahan Fertility and Infertility Center were initially assessed according to World Health Organization (WHO) criteria. Samples were washed twice with Ham’s. Each sample was divided into two portions, a control and the other processed for density gradient centrifugation (DGC). Each portion was assessed for CMA3 staining by both the slide and flow cytometry methods. Coefficients of correlation and student t-test were carried out using the Statistical Package for the Social Studies (SPSS 11.5).

**Results:**

Detection of CMA3 staining was more appropriate with fluorescence detector 3 (FL-3) rather than fluorescence detector 2 (FL-2) in the evaluation of protamine deficiency to exclude M540 bodies.

**Conclusion:**

This study, for the first time, provides the basis for assessment of CMA3 staining for flow cytometry. However, since the maximum excitation for CMA3 is not covered by the 488 nm laser, we recommend further experimentation using a flow cytometer with optimal excitation.

## Introduction

Daily exposure to a number of common chemicals in the home and workplace is one of the main reasons for infertility. Both environmental and other causes have made infertility a pandemic phenomenon. Therefore, understanding the precise molecular mechanisms underlying infertility may provide useful hints for its prevention and treatment (1).

Assessment of male infertility based upon semen analysis as described by the World Health Organization (WHO) guidelines only provides quantitative and qualitative analysis rather than mechanistic or functional information (2). In this regard, sperm function tests are becoming an inevitable part of the andrology laboratory. The clinical application of each test is being evaluated. Tests such as the sperm chromatin structural assay (SCSA), sperm chromatin dispersion test and TUNEL assay are becoming more common as complementary tests for semen analysis. The two former tests are based on the susceptibility of sperm to undergo denaturation while the latter assesses sperm DNA fragmentation (3-5).

One of the main factors affecting the susceptibility of sperm to undergo denaturation and inevitably DNA damage is the proper replacement of histones with protamine during chromatin compaction in the process of spermiogenesis (6). Protamine efficiency can be assessed indirectly by chromomycin A3 (CMA3), which has been shown to compete with protamine to bind DNA (7-9). Following the first report on the use of CMA3 for assessment of protamine deficiency by Bianchi et al. in 1993 there have been over 1500 reports regarding the implementation of CMA3 staining for infertility assessment. These techniques have been based on fluorescence microscopy (10). Flow cytometry, compared to fluorescence microscopy or the slide method has many advantages and a few disadvantages. The advantages of flow cytometry include analysis of thousands of cells within a few seconds, providing a statistically more precise evaluation by a reproducible technique, reducing intra-observer variations and sperm counts based on DNA staining. One disadvantage is the absence of direct observation (11).

In a previous study, we standardized the CMA3 staining procedure to indirectly assess semen sample protamination by flow cytometry. Our study determined that sperm could be assessed as fixed or unfixed. The fluorescence microscopic procedure utilized washed sperm samples that were fixed prior to assessment. According to our research; both the sperm concentration and duration of exposure should be constant. Thus, we have proposed that a concentration of 2 million sperm should be stained with CMA3 solution for an optimal time of 60 minutes. Fixed samples can be stained and assessed later (12).

A study of the literature has revealed that semen in addition to somatic and germ cells may contain round structures like sperm head, in different dimensions, and are observable via light microscopy. They are apoptotic bodies which have been named merocyanine 540 (M540) bodies, since they promptly stain with M540 and contain no DNA or small quantities of fragmented DNA (13). Therefore, M540 bodies are not labeled with nuclear probes, such as propidium iodide (PI) or Yo-Pro-1.

These properties partially locate these M540bodies in the same region of flow cytometric dot plot (forward and side scatter) that contain sperm samples. Therefore, if M540 bodies are not excluded from flow cytometric analysis the results will be underestimated (14-16). Muratori et al. have shown that due to the heterogeneous nature of M540, in terms of size and density, they migrate differently during the density gradient centrifugation (DGC) of sperm. Thus, M540 are completely or partially isolated during the swim up and DGC procedures used for sperm processing (17).

CMA3 is a flurochrome that can be assessed by both fluorescence detectors 2 (FL-2) and 3 (FL-3) and to our knowledge, the choice of detector used when staining with CMA3 remains to be determined. Therefore, the aim of this study is to evaluate the percentage of CMA3 by PI staining with the purpose of excluding M540 bodies as they prevent underestimation of the percentage of CMA3 positivity by flow cytometery.

## Materials and Methods

### Sperm analysis and processing

Semen samples were collected from 104 infertile men who referred to the Andrology Unit of the Isfahan Fertility and Infertility Center. This experimental study was approved by Royan Institutional Review Board. Informed consent forms were signed by all the individuals who provided semen samples for this study. Samples were initially assessed according to WHO criteria (2) and then washed twice with Ham’s at 400g centrifugation for 5 minutes prior to being separated into two portions. One portion was used as a control while the other was processed for DGC using PureSperm (Nidacon, Gothenburg, Sweden) according to the manufacturer’s specifications. Each portion was assessed for CMA3 staining by both the slide and flow cytometric methods.

### Assessment of M540 bodies by merocyanine and Yo-Pro-1

For measurement of M540 bodies, 15μl from M540 (2.09 μM; Sigma, USA) and Yo-Pro-1 (0.01 μM; Sigma, USA) were added to the sperm suspension and incubated at room temperature for 20 minutes in the dark. For sample analysis, a FACS Calibur flow cytometer (Becton Dickinson, San Jose, CA, USA) equipped with a 488 nm laser was chosen and 10000 apparently sperm-specific events were calculated. A forward and side scatter (FSC and SSC) gate was used to select single sperm from debris and aggregates. Fluorescence compensation was set by unstained sperm and separately stained with Yo-Pro-1 and M540. Green fluorescence from Yo-Pro-1 and red fluorescence from M540 were collected in fluorescence detector 1 (FL-1) with a 530/30 nm band-pass filter and FL-2 with a 585/42 nanometer (nm) band-pass filter, respectively. Data were analyzed using BD Cell Quest Pro and WinMDI 2.9 software.

### Assessment of protamine deficiency (chromomycin A3 staining)

CMA3 staining was assessed according to a previously described procedure for CMA3 flow cytometry and fluorescence microscopy (7, 12). Briefly, the control and DGC processed samples were fixed with Cranoy’s solution and divided into three portions. One portion was used to prepare the smear for fluorescence microscopy, which was stained for 20 minutes with 100 μl of 0.25 mg/ml CMA3 solution (Sigma, USA). For flow cytometry, the concentration of sperm was adjusted to 2 million per ml, centrifuged and fixed with Cranoy’s solution. The pellet was resuspeded in 200 μl of 0.25 mg/ml of CMA3 solution for 1 hour. Then, samples were washed and used for flow cytometry and assessed by FL-2 and FL-3. To 500 μl of the third portion, which contained 1 million fixed sperm, 5 μl PI (1 μg/ml) was added for 10 minutes before assessment with flow cytometry.

The excitation range of CMA3 has been reported to be between ~350 and ~500nm (maximum excitation limitation: 430-457 nm). In this study we use a 488 nm laser, which is at the end of the excitation range of CMA3 that allowed us to obtain detectable emissions in both FL-2 and FL-3. Therefore, we recommend the results to be repeated on a flow cytometer equipped with a laser to emit light at around 445 nm (between 430 to 457 nm) to excite CMA3 (18, 19).

### Calculation of CMA3 based on PI staining

Following assessment of CMA3 by flow cytometry, the number of CMA3 positive sperm in 10000 counted cells was defined. This number was divided by the number of PI positive cells in the 10000 counted cells, multiplied by 10000 cells with the assumption that all the counted CMA3 positive cells were sperm. For example, the number of CMA3 positive cells was 30% or 3000 in 10000 counted cells and the number of PI positive cells was 95% or 9500 cells in the 10000 counted cells. Therefore, the true number of CMA3 positive cells would be 3160 rather than 3000 cells per 10000 counted and the true percentage of CMA3 would be 31.6% instead of 30%.

### Statistical analysis

Results are expressed as means ± SEM. For statistical analysis, coefficients of correlation and paired t-tests were carried out using the Statistical Package for the Social Studies (SPSS, version. 11.5, Chicago, IL, USA) software to compare results between different groups. A probability of p<0.05 was considered statistically significant.

## Results

**Fig 1:**
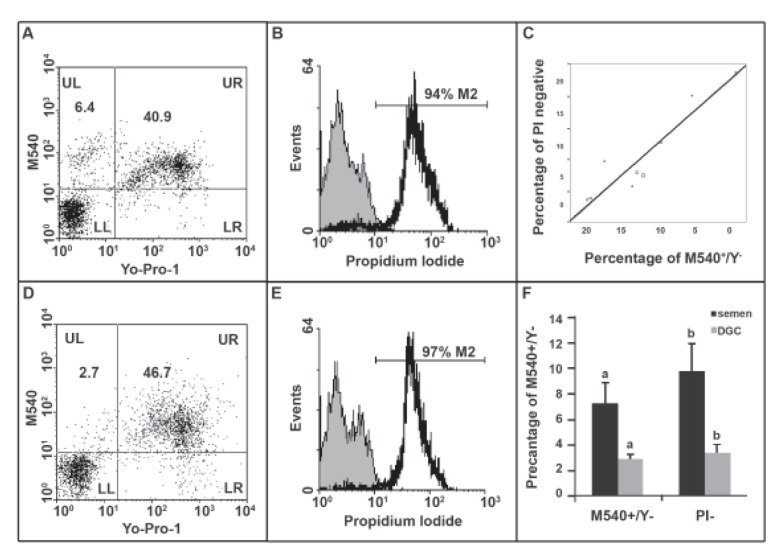
(A) Dot plot of M540 versus Yo-Pro-1 staining and (B) PI histogram in a semen sample. (C). Correlation between the percentage of PI negative cells and M540 positive/ Yo-Pro-1 negative. (D) Dot plot of M540 versus Yo-Pro-1 staining and (E) PI histogram in a processed sample by DGC. (F) Comparison of the percentage of PI negative cells and M540 positive/ Yo-Pro-1 negative before and after processing. Common letters are significantly different at p<0.05.

Figure 1A shows the plot of M540 and Y1 staining in semen samples (upper left are apoptotic bodies, or the M540 positive, and Y1 negative) while Figure 1B shows the PI histogram of the same sample (PI negative population). We assessed the correlation between the percentage of PI negative cells and the percentage of M540 bodies in ten semen samples where a strong correlation was observed (r=+0.95, p<0.001, Fig 1C). These samples were further processed with DGC and the percentage of M540 bodies was assessed by both M540/Y1 (Fig 1D) and with PI (Fig 1E). The results of Figure 1F show that DGC has isolated these M540 bodies as assessed by both M540/Y1 and PI staining in the same ten semen samples. In the remaining experiments we assessed only PI as an indication for M540 bodies.

**Fig 2:**
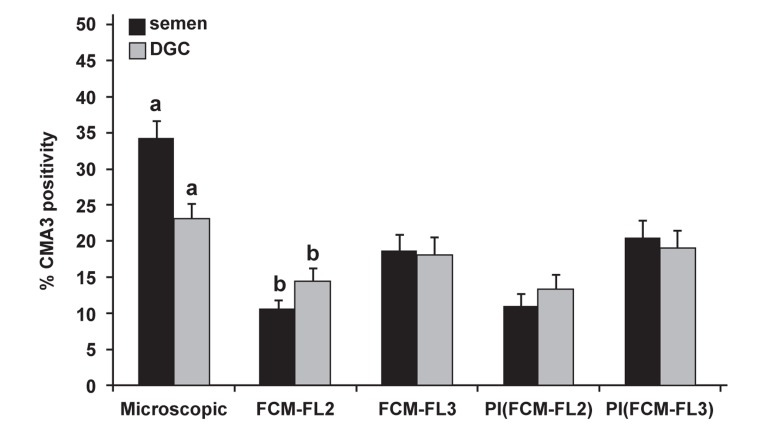
Comparison of the percentage of sperm with CMA3 positivity by fluorescence microscopy and flow cytometry using fluorescence detectors 2 or 3 (FL-2 and FL-3) in semen and DGC. Common letters are significantly different at p<0.05.

Following the above conclusion, 104 samples were simultaneously assessed for CMA3 staining by fluorescence microscopy and flow cytometry by FL-2 and FL-3. Of the samples, 86 were processed simultaneously by DGC and then assessed for CMA3 staining. Both the processed and neat samples have been assessed for the percentage of PI positive or negative cells. Figure 2 shows that in the DGC procedure, the percent of the CMA3 positive sperm decreased significantly with fluorescence microscopy. However assessment by flow cytometry revealed an increase in the percentage of CMA3 positive cells post-DGC, when FL-2 was used and a decrease in the percentage of CMA3 positive cells when FL-3 was implemented, irrespective of apoptotic bodies (with and without PI). These values were only significantly different when FL-2 was implemented without taking into account the M540 bodies.

**Fig 3:**
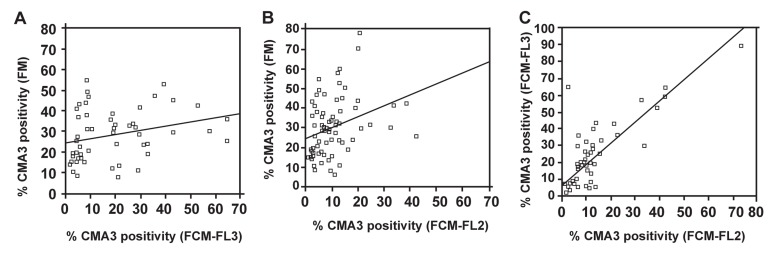
Correlations of the percentage of CMA3 positivity assessed by fluorescence microscopy and flow cytometry using fluorescence detector 2 or 3 (FL-2 and FL-3).

Figures 3A and B show the correlations of percent of CMA3 positivity as assessed by fluorescence microscopy and flow cytometry using FL-2 and FL-3. A significant weak correlation was observed between fluorescent microscopy and flow cytometry using FL-2 (r=0.320; p=0.006) and FL-3 (r=0.273; p=0.048). However, a strong significant correlation was observed between FL-2 and FL-3 (r=0.819 and p<0.001, Fig . 3C).

**Fig 4:**
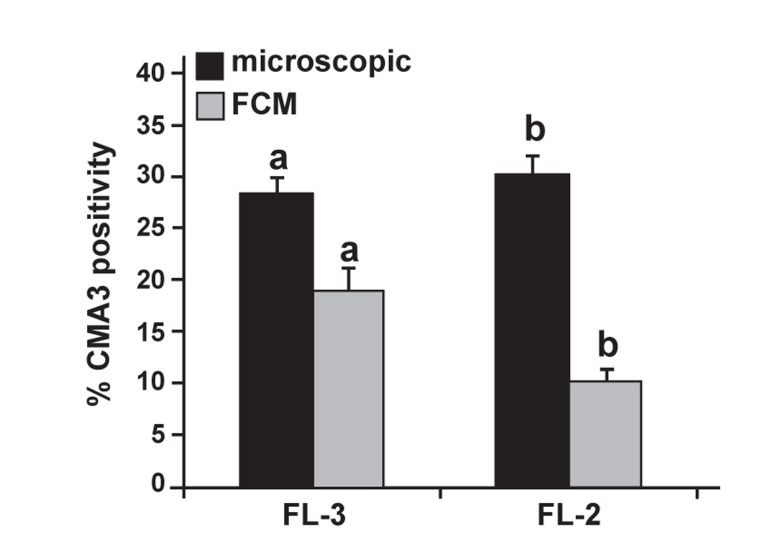
Comparison of the percentage of sperm with CMA3 positivity between the slide method and flow cytometry using fluorescence detectors 2 or 3 (FL-2 and FL-3). Common letters are significantly different at p<0.05.

In addition, a comparison of the mean percent of CMA3 between the slide method and flow cytometry using FL-2 or FL-3 showed that both flow cytometric measurements were significantly lower than the slide method. However, the mean FL-3 measurement was closer to the slide method (Fig 4).

## Discussion

The presence of over 1500 reports in the literature emphasizes the evaluation of protamine content by indirect CMA3 staining. Except for our recent report on standardization and implementation of CMA3 staining by flow cytometry in sperm (12), there are few reports which have used CMA3 with flow cytometry based on the microscopic or slide method, as initially introduced by Bianchi et al. in 1993 (10). In this and our previous study we have shown that the mean percentage positive value of CMA3 as assessed by flow cytometry was lower than the slide method. Additionally the mean values were lower when assessed by FL-2 than FL-3 (Fig 4). One possible explanation that may account for this difference could be the underestimation of the percent of CMA3positive sperm due to the presence of M540 bodies in semen. Therefore, in order to exclude these bodies from our evaluation and provide an easy procedure to account for underestimation, we initially evaluated the role of PI staining in semen samples prepared for CMA3 staining.

The most common procedure for the assessment of M540 bodies is the use of merocyanine along with Y1 staining (15). In this study, we have aimed to distinguish these bodies from sperm with PI staining since they contain no or minute amounts of DNA. A very strong correlation between the percentages of PI negative events with these bodies (M540 positive and Yo-Pro-1 negative) in flow cytometry was shown. This suggested that PI negative events in flow cytometry were most likely M540 bodies (Fig 1A, B, C). This was further verified by the fact that sperm processing reduced their numbers and in this study both M540 positive/ Yo-Pro-1 negative and PI negative events were reduced following sperm density gradient. Therefore, based on these data we provided an easy calculation method (as seen in the Materials and Methods section) to exclude M540 bodies from our sperm population during CMA3 assessment, which was based on PI staining of solely fixed semen samples.

The results of this study also revealed that the percentage CMA3 positive sperm obtained by the slide method or microscopic procedure, despite accounting for M540 bodies, were still significantly greater than the flow cytometric method, irrespective of assessment by FL-2 or FL-3 (Fig 4). As with our previous study, we obtained a very weak correlation between the slide method and flow cytometric procedure (Fig 3) (12). Although the slide method has been previously assessed for intra- and inter-observation variation with a low coefficient of variation (20) this difference may solely be contributed to instrumental precision. With the instrument, a threshold is described based on unstained samples while such precision cannot be visually attained. Indeed, a similar observation has been previously reported for acridine orange staining. Therefore the sperm chromatin structural assay which is a routinely accepted clinical test based on acridine orange staining is considered acceptable when carried out by flow cytometry and not by the slide method (21).

In flow cytometry the mean values of CMA3 obtained, excluding M540 bodies, are significantly greater in FL-3 than FL-2. Despite a significant correlation between the CMA3 assessment by FL-2 and FL-3, figure 3A and B show a wider range of CMA3 in FL-3 than FL-2; this may suggest that variations within a heterogeneous sample are more assessable in FL-3. In addition, as shown in this study and well accepted in the literature, one expects a reduction in the percentage of CMA3 positive sperm following DGC, even though no significance has been observed with FL-3, in contrast to FL2. Although we could not account for this difference between FL-2 and FL-3, despite the use of the same semen samples for these assessments, one possible explanation could be that FL-2 does not provide the optimal wavelength for assessment of CMA3.Therefore, variations within samples are more observable in FL-3.

## Conclusion

In considering the evaluation of CMA3 for assessment of protamine deficiency, we propose the assessment of CMA3 by flow cytometry using FL-3 and staining for PI to exclude M540 bodies, especially in samples with oligo-astheno-teratospermia or astheno-tratospermia where the presence of these bodies has been reported to be high (15). Therefore, evaluation of CMA3 staining as clinical tool by flow cytometry awaits further research. However, we recommend further experimentation using a flow cytometer with optimal excitation for CMA3.
